# Electromagnetic Imaging in Half-Space Using U-Net with the Iterative Modified Contrast Scheme

**DOI:** 10.3390/s25041120

**Published:** 2025-02-12

**Authors:** Chien-Ching Chiu, Ching-Lieh Li, Po-Hsiang Chen, Yen-Chun Li, Eng-Hock Lim

**Affiliations:** 1 Department of Electrical and Computer and Engineering, Tamkang University, New Taipei City 251301, Taiwan; chingliehli1001@gms.tku.edu.tw (C.-L.L.); 810440031@gms.tku.edu.tw (P.-H.C.); 6124440221@o365.tku.edu.tw (Y.-C.L.); 2 Department of Electrical and Electronic Engineering, University Tunku Abdul Rahman, Kajang 43200, Malaysia; limeh@utar.edu.my

**Keywords:** iterative modified contrast scheme, inverse scattering problem, U-Net, structural similarity

## Abstract

U-Net with the iterative modified contrast scheme (IMCS) is proposed to solve inverse scattering problems (ISPs) in half-space. IMCS is an innovative inversion technique that utilizes contrast functions to improve the visibility of target regions and reconstruct the internal structure of objects. In contrast to applying IMCS alone, our proposed method improves the detection of contrast boundaries, enhancing noise immunity as well as increasing the structural similarity (SSI) through deep learning with U-Net. We compare the numerical results for 200-iteration IMCS and U-Net with 3-iteration IMCS, and it is found that the accuracy of reconstructed images can be improved a lot by U-Net with the 3-iteration IMCS architecture. In addition, even in the case of large Gaussian noise, the reconstruction is still good with our proposed method.

## 1. Introduction

Electromagnetic imaging (EMI) uses electromagnetic waves to visualize internal structures by analyzing how materials absorb, reflect, or refract these waves. This technology has considerable potential for practical applications in the fields of medical imaging, non-destructive testing, and geophysical exploration in the future. In medical techniques such as Magnetic Resonance Imaging (MRI), X-rays, and CT scans, it is used to reveal tissues and bones. In geophysical applications, ground-penetrating radar is used to detect underground formations. EMI is also essential in industrial non-destructive testing (NDT) and security screening. Despite its wide applications, challenges remain, including balancing image resolution with penetration depth, managing interference, and ensuring safety, particularly with ionizing radiation. The most frequently applied image reconstruction methods are traditional optimization algorithms [1,2,3,4] and deep learning-based approaches [5,6,7,8,9,10,11,12,13,14]. Algorithms are categorized into two types: non-iterative [1,2,4] and iterative [3]. Non-iterative algorithms include the Born Approximation (BA), Distorted Born Approximation (DBA), and Rytov Approximation (RA). These algorithms can reconstruct images once a satisfactory approximation is achieved. Unfortunately, non-iterative algorithms are effective only for weak scatterers. Although iterative techniques, such as gradient descent and genetic algorithms, are capable of solving problems, they tend to be computationally intensive and less accurate when applied to complex electromagnetic imaging tasks.

Convolutional Neural Networks (CNNs) have proven highly effective in image processing, leveraging multi-layered feature extraction and large dataset handling [5,6,7,8,9,10,11,12,13,14]. CNNs have transformed tasks like image comparison, object classification, and image segmentation. Their architecture, inspired by the visual cortex, incorporates convolutional layers to detect spatial hierarchies of features, pooling layers for dimensionality reduction, and fully connected layers for decision-making. The key strength of CNNs lies in their ability to learn hierarchical representations of data. Early layers typically capture low-level features such as edges and textures, while deeper layers progressively identify more complex patterns like shapes and objects. This hierarchical feature extraction enables CNNs to generalize effectively across diverse datasets. Additionally, advancements such as transfer learning, data augmentation, and architectures like ResNet, EfficientNet, and MobileNet will further enhance efficiency and accuracy. However, deep learning techniques demand significant computational power for training and often struggle with generalization, especially when dealing with diverse data, which can result in overfitting. In 2019, Li et al. proposed a deep CNN architecture designed to enhance image reconstruction quality and efficiency for ill-posed, nonlinear electromagnetic ISPs [5]. They utilized a non-iterative, complex-valued CNN, which demonstrated impressive stability and generalization capabilities. The following year, Xiao et al. developed a rapid electromagnetic inversion technique for inhomogeneous objects in layered media, combining the Born Approximation with a 3D U-Net [6]. This approach resulted in significant improvements in both reconstruction accuracy and processing efficiency. Building on these advancements, Zhou et al. addressed global nonlinearity in ISPs by introducing a modified contrast method that yielded efficient and accurate real-time reconstructions in numerical tests in 2001 [7]. In 2022, Xu’s team further refined CNN-based reconstruction by using scalable cascaded networks, breaking down the process into linear transformations and multi-resolution imaging networks [8]. This approach significantly enhanced both efficiency and inversion accuracy. In 2023, Ma et al. introduced total variation regularization techniques incorporated with a deep learning neural network for ISPs [9]. This innovation effectively reduced background artifacts and enhanced the clarity of geometric features in reconstructions. Later in the same year, Hu et al. introduced a transceiver transformation module with a hybrid forward-inverse neural network to improve the quality of deep learning-based electromagnetic image technique [10]. This approach significantly reduced data misfit and efficiently reconstructed dielectric distributions across various frequencies and configurations. Recently, Chiu et al. proposed the use of a combined modified contrast scheme (MCS) technique to reconstruct microwave imaging of uniaxial objects in free space. In comparison with the DCS, MCS outperforms DCS in the reconstruction of the dielectric constant when the dielectric constant is large [11].

Regarding the papers for the half-space electromagnetic image recently, in 2023, T. Oğuz Topbaş established a classical moment method using vertical coordinates to find a numerical solution to the two-dimensional backscattering problem associated with a cylindrical body buried in a half-space of known orientation [12]. In 2024, Chiu et al. proposed a new artificial intelligence method incorporating hemispheric electromagnetic imaging attention mechanisms. The method first generates an initial guess image from the measured scattering field of a buried scatterer in the lower hemisphere using a BPS. This initial guess image is then fed to a generative actuarial network (GAN) and a self-attentive generative adversarial network (SAGAN) for further processing [13]. To the best of our knowledge, there is no research on U-Net with the IMCS technique for the half-space electromagnetic imaging.

**Figure 1:**
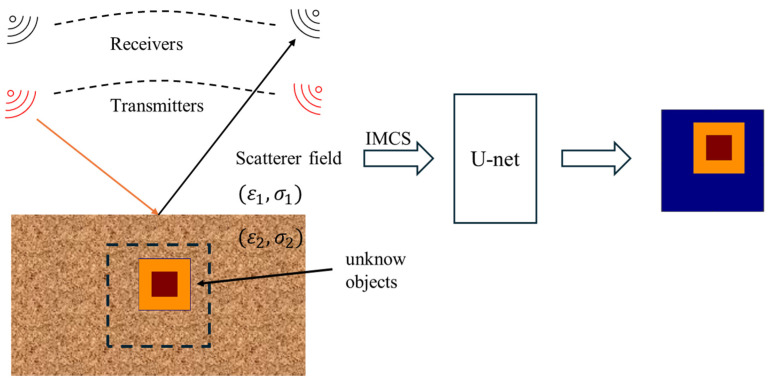
Sensing and reconstruction architecture.

This study uses a half-wave dipole antenna to transmit microwave signals to detect unknown objects located in half-space by sensing the scattered field at the receivers, as shown in Figure 1. First, IMCS is used to generate a preliminary image from the sensed scattered field. This initial image is then input into the U-Net model for enhanced reconstruction, resulting in a high-resolution electromagnetic image. The key contributions of this article are as follows:

(1) U-Net with IMCS was used to reconstruct high-contrast scatterers. IMCS improves the contrast of the initial input image in an iterative manner, which can provide a better preliminary image for U-Net and deal with complex high-contrast objects.
(2) IMCS enhances image contrast and highlights target features more clearly. The combination of IMCS and U-Net, which utilizes U-Net for fine segmentation of the enhanced image, improves the accuracy and stability of image processing.
(3) Compared with [11], which only deals with free-space cases, our proposed U-Net with IMCS is first presented for buried object detection.

This paper is organized as follows. Section 1 is the introduction. Section 2 discusses the forward representation of buried objects and the theory of IMCS. Section 3 presents the overall flow charts and structure of U-Net. Section 4 provides numerical results. Section 5 is the conclusion.

## 2. Theory

### 2.1. Forward Problem

**Figure 2:**
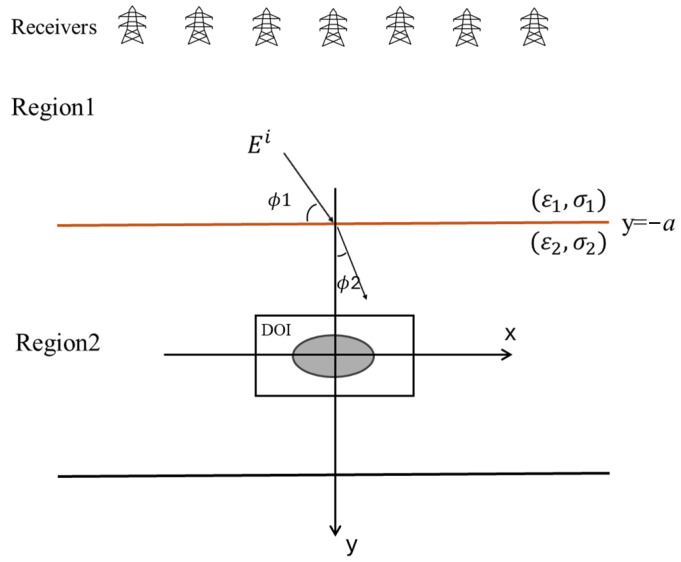
Classical configuration of buried objects in the plane.

Figure 2 describes a dielectric (non-conductive) object situated in a lossy half-space, where the space can conduct electricity with some resistance. We have two regions in this space. In Regions 1 and 2, the permittivity and conductivity are $\varepsilon_{1}$, $\sigma_{1}$ and $\varepsilon_{2}$, $\sigma_{2}$, respectively. Both regions have the same magnetic permeability as free space, denoted by $\mu_{0}$. This means that we only consider non-magnetic materials. The object is illuminated by an incident transverse magnetic (TM) wave, i.e., the electric field is perpendicular to the direction of wave propagation, while the magnetic field has components in both the radial and axial directions. The incident wave has a time-harmonic nature, meaning its behavior over time can be expressed with a sinusoidal function $e^{j\omega t}$ and hits the object at an angle $\phi_{1}$. This representation reflects the oscillating nature of the electromagnetic field.

To simplify the analysis, we assume that the electric field of the TM wave is polarized along the *z*-axis. In this case, if there is no scatterer in the space, the electric field of the incident wave can be expressed as $E^{i}$ in the following mathematical form:

(1) $$\begin{array}{l} \bar{E^{i}}\left(\vec{r}\right)=E^{i}\left(x,y\right)\hat{z}\\ =\left\{\begin{array}{l} E_{1}\left(x,y\right)=e^{-jk_{1}[x{\mathrm{sin\phi}}_{1}+(y+a){\cos\phi }_{1}]}+R_{1}e^{-jk_{1}[x{\mathrm{sin\phi}}_{1}-(y+a){\mathrm{cos\phi}}_{1}]},y\leq -a\\ E_{2}\left(x,y\right)=Te^{-jk_{2}[x{\mathrm{sin\phi}}_{2}+(y+a){\mathrm{cos\phi}}_{2}]}\;\;\;\;\;\;\;\;\;\;\;\;\;\;\;,y>-a \end{array}\right. \end{array}$$

where

(2) $$R_{1}=\frac{1-n}{1+n},\;\;T=\frac{2}{1+n},\;\;n=\frac{{cos\phi }_{2}}{{cos\phi }_{1}}\sqrt{\frac{\varepsilon_{2}-j\sigma_{2}/\omega }{\varepsilon_{1}-j\sigma_{1}/\omega }}$$

(3) $$k_{1}sin\phi_{1}=k_{2}sin\phi_{2}$$

(4) $$k_{i}^{2}=\omega^{2}\varepsilon_{i}\mu_{0}-j\omega \mu_{0}\sigma_{i}\;\;,\;\;i=1,2\;\;Im(k_{i})\leq 0$$

If Regions 1 and 2 are composed of lossless media, $\phi_{1}$ and $\phi_{2}$ correspond to the propagation directions of incident and refracted waves, respectively, and can be described by simple geometrical relations. However, when Regions 1 and 2 are composed of lossy media, the angles $\phi_{1}$ and $\phi_{2}$ is no longer a simple real number but a complex number because the wave decay and phase change need to be considered. At this point, the propagation directions of incidence and refraction, as well as the energy decay of the wave in the medium, will become more complex and need to be described by wave vectors in complex form.

Based on the concept of induced current and Maxwell’s equations, we can derive the following equations:

(5) $$\nabla \times \bar{E}=-j\omega \mu_{0}\bar{H}$$

(6) $$\nabla \times \bar{H}=j\omega \varepsilon_{2}\bar{E}+{\overset{⃑}{j}}_{eq}$$

where ${\overset{⃑}{j}}_{eq}=j\omega \varepsilon_{0}\left[\varepsilon_{ob}\left(x,y\right)-\varepsilon_{2}\right]E\hat{z}$ is the equivalent current density of the dielectric object, $\varepsilon_{r}=\frac{\varepsilon_{ob}}{\varepsilon_{2}}$ is the relative permittivity to $\varepsilon_{2}\;,\;\varepsilon_{ob}$ is the permittivity of the object.

The total electric field inside the object $\bar{E^{t}}\left(x,y\right)=E^{t}\left(x,y\right)\hat{z}=\left[E^{i}\left(x,y\right)+E^{s}\left(x,y\right)\right]\hat{z}$ can be expressed by the two-dimensional Green’s function as follows:

(7) $$E^{i}\left(\bar{r}\right)=\int_{D}G(x,\;y;\;x\prime ,\;y\prime )k_{2}^{2}\left[\varepsilon_{r}\left(\left(x\prime ,\;y\prime \right)\right)-1\right]E^{t}\left(\left(x\prime ,\;y\prime \right)\right)ds^{\prime }+E^{t}\left(x,\;y\right),\;\;y>-a$$

The scattered field can be written as follows:

(8) $$E^{s}\left(\bar{r}\right)=-\int_{D}G\left(x,\;y;\;x^{\prime },\;y^{\prime }\right)k_{2}^{2}\left[\varepsilon_{r}\left(\left(x^{\prime },\;y^{\prime }\right)\right)-1\right]E^{t}\left(\left(x^{\prime },\;y^{\prime }\right)\right)ds^{\prime }+E^{t}\left(x,\;y\right),\;\;y>-a$$

To address this half-space problem, the Green’s function, denoted as G(x, y; x′, y′), must first be determined. This process involves introducing a line current source located at (x′, y′) and calculating the resulting scattered field observed at (x, y). By applying the Fourier transform technique, the half-space Green’s function G(x, y; x′, y′) can be reformulated and represented as follows:

(9) $$G\left(x,y{;x}^{\prime },y^{\prime }\right)=\left\{\begin{array}{l} G_{1}\left(x,y{;x}^{\prime },y^{\prime }\right)\;\;\;\;\;\;\;\;\;\;\;\;\;\;,\;y\leq -a\\ G_{2}\left(x,y{;x}^{\prime },y^{\prime }\right)=G_{f}\left(x,y{;x}^{\prime },y^{\prime }\right)+G_{y^{\prime }}\left(x,y{;x}^{\prime },y^{\prime }\right)\;\;\;\;,y>-a \end{array}\right.$$

(10) $$G_{1}\left(x,y{;x}^{\prime },y^{\prime }\right)=\frac{1}{2\pi }\int_{-\infty }^{\infty }\frac{j}{\gamma_{1}+\gamma_{2}}e^{j\gamma_{1}\left(y+a\right)}e^{-j\gamma_{2}\left(y\prime +a\right)}e^{-j\alpha \left(x-x^{\prime }\right)}d\alpha$$

(11a) $$G_{f}\left(x,y{;x}^{\prime },y^{\prime }\right)=\frac{j}{4}H_{0}^{\left(2\right)}\left[k_{2}\sqrt{(x-x\prime {)}^{2}+(y-y\prime {)}^{2}}\right]$$

(11b) $$G_{s}\left(x,y{;x}^{\prime },y^{\prime }\right)=\frac{1}{2\pi }\int_{-\infty }^{\infty }\frac{j}{2\gamma_{2}}\left(\frac{\gamma_{2}-\gamma_{1}}{\gamma_{2}+\gamma_{1}}\right)e^{-j\gamma_{2}\left(y+2a+y^{\prime }\right)}e^{-j\alpha \left(x-x^{\prime }\right)}d\alpha$$

(12) γi2=ki2−α2,  i=1,2 ,  Im⁡(γi)≤0

In this context, $k_{i}$ represents the wave number of the *i*-th region, and G(x, y; x′, y′) denotes the half-space Green’s function, which is obtained through the Fourier transform in Equation (9), where $H_{0}^{2}$ refers to the second-order zero Hankel function. When solving Equations (7) and (8) numerically, it is essential to compute the Green’s function from Equation (9) in advance. However, when the points (x, y) and (x′, y′) get very close to the interface between the two regions at y=−a, the integration process becomes slow to converge. As a result, this significantly increases the computational load when calculating the half-space Green’s function in such situations. The forward problem for Equations (7) and (8) can be written in two formulas:

(13) $$E^{t}\left(x,y\right)=E^{i}\left(x,\;y\right)+\int_{D}G^{2D}\left(x,y;x^{\prime },\;y^{\prime }\right)I\left(x^{\prime },\;y^{\prime }\right)ds\prime \;,\;y>-a$$

(14) $$E^{s}\left(x,y\right)=\int_{D}G^{2D}\left(x,y;x\prime ,\;y\prime \right)I\left(x\prime ,\;y\prime \right)ds\prime ,\;y<-a$$

Equation (13) is the field equation. $E^{t}$ denotes the total electric field. $G^{2D}$ is the Green’s function for 2D and equal to $-{k_{2}}^{2}G$. The induced contrast current (ICC) *I* (x,y) in D is defined as $I(x,y)=\chi (x,y)E^{t}(x,y)$, with the contrast *χ* (x,y) = $\varepsilon_{r}$(x,y) − 1. Equation (14) is the data equation. $E^{s}$ denotes the scattered electric field on the measurement domain. As in [15], a modified contrast $R\left(x,y\right)=\beta \left(x,y\right)\chi \left(x,y\right){\left[\beta \left(x,y\right)\chi \left(x,y\right)+1\right]}^{-1}$ is proposed to multiply both sides of (13), then the contraction integral equation for inversion can be obtained:

(15) $$\beta \left(x,y\right)I\left(x,y\right)=R\left(x,y\right)\beta \left(x,y\right)I\left(rx,y\right)+R\left(x,y\right)\left[E^{i}\left(x,y\right)+\int_{D}G^{2D}\left(x,y;x\prime ,\;y\prime \right)I\left(x\prime ,\;y\prime \right)ds\prime \right]\;,for\;x,y\in D$$

In this context, β acts as a local-wave amplifier, intensifying the effect of the introduced term $R\left(x,y\right)\beta \left(x,y\right)I\left(x,y\right)$. To distinguish this term from the global term $R\left(r\right)\int_{D}G^{2D}\left(x,y;x\prime ,\;y\prime \right)I\left(x\prime ,\;y\prime \right)ds\prime$ in (15), it is referred to as the local term, as the induced current at a given position depends only on that position and not on others. In comparison to (13), where the local term is $E^{i}\left(x,y\right)$, and the global term is $\int_{D}G^{2D}\left(x,y;x^{\prime },\;y^{\prime }\right)I\left(x^{\prime },\;y^{\prime }\right)ds\prime$, (15) includes a greater proportion of the local term, which is influenced by the value of *β*. A larger *β* value amplifies the local effect.

### 2.2. Iteration Procedure in MCS

The goal of ISPs is to reconstruct or infer the properties of an unknown object and medium from measured scattered data. This typically involves determining the shape, structure, composition, or material properties of the target based on how it interacts with incident waves. Such an optimization procedure can be formulated as follows:

(16) $$Min:h\left(\varepsilon_{r}\right)=\sum_{P=1}^{N_{i}}{\left\|Q\left(E_{p}^{i},\varepsilon_{r}\right)-E_{p}^{s}\right\|}^{2}+\alpha T\left(\varepsilon_{r}\right)$$

Let Q represent the forward problem solver, T denote the regularization function, and α be the constant regularization coefficient; $N_{i}$ is the number of incidences. The DOI is divided into M×M subunits, enabling the application of the method of moments (MOM) with pulse basis functions and delta test. The center of each subunit is located at $r_{n}$, $n=1,\;2,\;\ldots ,\;M^{2}$. After discretization, the discretized form of (14) and (15) is obtained:

(17) $${\bar{E}}^{s}={\overset{̿}{G}}_{s}^{2D}\cdot \bar{I}$$

(18) $$diag\left(\bar{\beta }\right)\cdot \bar{I}=diag\left(\bar{R}\right)\cdot \left[diag\left(\bar{\beta }\right)\cdot \bar{I}+{\bar{E}}^{i}+{\overset{̿}{G}}_{D}^{2D}\cdot \bar{I}\right]$$

where $\bar{\beta }$, $\bar{R}$, $\bar{I}$, and ${\bar{E}}^{i}$ are $M^{2}\times 1$ vectors, and diag(⋅) is the operator that returns a square diagonal matrix with the elements of the vector on the main diagonal. ${\overset{̿}{G}}_{D}^{2D}$ is an $M^{2}\;$× $M^{2}$ matrix that maps the ICC to the scattered field in Region 2, and ${\overset{̿}{G}}_{s}^{2D}$ is an $N_{r}\times M^{2}$ matrix that maps the ICC to the scattered field in Region 1. The ICC $\bar{I}$ in (17) and (18) can be divided into two parts, located in two orthogonal and complementary subspaces spanned by the singular vectors of ${\overset{̿}{G}}_{s}^{2D}\;$ [16]. One is the dominant part of the induced current (DPIC) ${\bar{I}}^{d}$ encompassed by the expanse of prominent singular values, whereas the counterpart exists as the ambiguous part of the induced current (APIC) ${\bar{I}}^{a}$ within small and null singular value subspace. In other words, $\bar{I}={\bar{I}}^{d}+{\bar{I}}^{a}$. The outcome of performing a singular value decomposition on ${\overset{̿}{G}}_{s}^{2D}$ is that ${\overset{̿}{G}}_{s}^{2D}$ = $\Sigma_{n}{\bar{u}}_{n}\sigma_{n}{\bar{v}}_{n}^{H}$, where ${\bar{u}}_{n}$ represents the *n*-th left singular vector, $\sigma_{n}$ represents the *n*-th singular value, and ${\bar{v}}_{n}$ represents the *n*-th right singular vector, with the superscript *H* representing the Hermitian operator. Utilizing the orthogonality of the singular vectors, the DPIC ${\bar{I}}^{d}$ can be computed using the first *L* large singular values as follows:

(19) $${\bar{I}}^{d}=\sum_{n=1}^{L}\frac{\left({\bar{u}}_{n}^{H}\cdot \;{\bar{E}}^{s}\right)}{\sigma_{n}}\;{\bar{v}}_{n}$$

The choice of *L* is based on observing the characteristic value $\sigma_{n}$ using a logarithmic scale. This selection captures the maximum slope, making it the optimal value for *L*. During the process, only the singular values and vectors of the first *L* terms are considered for computation, significantly reducing the computational cost. In our case, we set *L* to 14. Unlike utilizing the fast Fourier transform twofold subspace-based optimization method (FFT-TSOM) to generate the APIC ${\bar{I}}^{a}$ mentioned in [16], we choose the APIC ${\bar{I}}^{a}$ itself as the unknown. This modification offers several advantages. Firstly, it involves generating the appropriate input for the neural network. By implementing only three iterations to minimize the objective function, we prefer using the APIC directly instead of relying solely on low-frequency Fourier bases, as in FFT-TSOM. This approach also shortens the iteration process of MCS, as it eliminates the APIC generation step in FFT-TSOM. By replacing $\bar{I}$ in (17) and (18) by ${\bar{I}}^{d}$ and ${\bar{I}}^{a}$, we can obtain a cost function for ${\bar{I}}_{p}^{a}$ for *p*-th incidence and $\bar{R}$:

(20) $$f\left({\bar{I}}_{1}^{a},{\bar{I}}_{2}^{a},\ldots {\bar{I}}_{N_{i}}^{a},\bar{\bar{R}}\right)=\sum_{p=1}^{N_{i}}\left[\frac{{\left\|\bar{\bar{A}}\cdot {\bar{I}}_{p}^{a}+{\bar{B}}_{p}\right\|}^{2}}{{\left\|{\bar{E}}_{p}^{i}\right\|}^{2}}+\frac{{\left\|{\bar{\bar{G}}}_{s}^{2D}\cdot {\bar{I}}_{p}^{a}+{\bar{\bar{G}}}_{s}^{2D}\cdot {\bar{I}}_{p}^{d}-{\bar{E}}_{p}^{s}\right\|}^{2}}{{\left\|{\bar{E}}_{p}^{s}\right\|}^{2}}\right]\;$$

where

(21a) $$\bar{\bar{A}}=diag\left(\bar{\beta }\right)-diag\left(\bar{R}\right)\cdot \left[diag\left(\bar{\beta }\right)+{\overset{̿}{G}}_{D}^{2D}\right]$$

(21b) $${\bar{B}}_{P}=diag\left(\bar{\beta }\right)\cdot {\bar{I}}_{p}^{d}-diag\left(\bar{R}\right)\cdot \left[diag\left(\bar{\beta }\right)\cdot {\bar{I}}_{p}^{a}+{\overset{̿}{G}}_{D}^{2D}\cdot {\bar{I}}_{p}^{a}+{\bar{E}}_{p}^{i}\right]$$

The noniterative back-propagation (BP) method can provide trustworthy reconstructed contrast when scatterers within *D* are weak. As the strength of the scatterers increases, the BP results increasingly deviate from the real contrast, sometimes even leading to misleading conclusions. To counter this, the preliminary estimate for the scatterer profile is set to be a homogeneous background medium. The Polak–Ribière conjugate gradient method is then employed to update the APIC ${\;\bar{I}}^{a}$ and the modified contrast $\bar{R}$ in an alternating fashion.

The goal of optimizing the objective function (20) is to produce a more precise input of the neural network. To save time during both the training and testing phases, only a few iterations are necessary. The implementation steps are as follows: First, select the background medium as the preliminary estimate of ${\bar{R}}_{0}$. Then, set ${\bar{I}}_{p,0}^{a}$ = 0 and the search direction ${\bar{\rho }}_{p,0}$ = 0. Second, update ${\bar{I}}_{p,n}^{a}$ with linear search method. Calculate gradient ${\bar{g}}_{p,n}=\nabla_{{\bar{I}}_{p}^{a}}f$ evaluated at ${\bar{I}}_{p,n-1}^{a}$ and ${\bar{R}}_{n-1}$:

(22) $$\nabla_{d}f={\bar{g}}_{p,n}=\frac{{\left[{\overset{̿}{G}}_{s}^{2D}\right]}^{H}\cdot \;\left({\overset{̿}{G}}_{s}^{2D}\right)\left({\bar{I}}_{p,n-1}^{a}\right)-{\bar{E}}_{p}^{s})}{{\left\|{\bar{E}}_{p}^{s}\right\|}^{2}}+\frac{{\overset{̿}{A}}^{H}\cdot \left[\;\overset{̿}{A}\;{\cdot \;\bar{I}}_{p,n-1}^{a}+{\bar{B}}_{p}\right]}{{\left\|{\bar{E}}_{p}^{i}\right\|}^{2}}$$

Calculate the search direction as follows:

(23) $${\bar{\rho }}_{p,n}={\bar{g}}_{p,n}+\frac{Re[{\left({\bar{g}}_{p,n}-{\bar{g}}_{p,n-1}\right)}^{H}\;\cdot \;{\bar{g}}_{p,n}]}{{\left\|{\bar{g}}_{p,n-1}\right\|}^{2}}{\bar{\rho }}_{p,n-1}$$

The step size $d_{p,n}$ for the *equation* ${\;\bar{I}}_{p,n}^{a}={\;\bar{I}}_{p,n-1}^{a}+d_{p,n}{\bar{\rho }}_{p,n}$ can be computed as the minimizer of (20). In other words, $d_{p,n}=Num/Den$. The numerator and denominator are as follows:

(24) $$Num=-{\left({\bar{\rho }}_{p,n}\right)}^{H}\cdot \;{\bar{g}}_{p,n}\;,Den=\frac{{\left\|{\overset{̿}{G}}_{s}^{2D}\cdot {\bar{\rho }}_{p,n}\right\|}^{2}}{{\left\|E_{p}^{s}\right\|}^{2}}+\frac{{\left\|\overset{̿}{A}\;\cdot {\bar{\rho }}_{p,n}\right\|}^{2}}{{\left\|E_{p}^{i}\right\|}^{2}}$$

Third, update ${\bar{R}}_{n}$. After updating the induced contrast current, ${\bar{I}}_{p,n}={\bar{I}}^{d}+{\;\bar{I}}_{p,n}^{a}$, the total field ${\bar{E}}_{p,n}^{t}$ can be refreshed.

(25) $${\bar{E}}_{p,n}^{t}={\bar{E}}_{p}^{i}+{\overset{̿}{G}}_{D}^{2D}\left({\bar{I}}_{p,n}\right)$$

The function takes a quadratic form with respect to ${\bar{R}}_{n}$ for the *m*-th cell, and the way to solve it is as follows:

(26) $${\bar{R}}_{n}\left(m\right)=\frac{\sum_{j}\left({\bar{I}}_{p,n}\cdot \bar{\beta }\right)\cdot {\left[\bar{\beta }\cdot {\bar{I}}_{p,n}+{\bar{E}}_{p,n}^{t}\;\right]}^{*}}{{\left|\bar{\beta }\cdot {\bar{I}}_{p,n}+{\bar{E}}_{p,n}^{t}\right|}^{2}}$$

where * denotes the conjugate operation.

**Figure 3:**
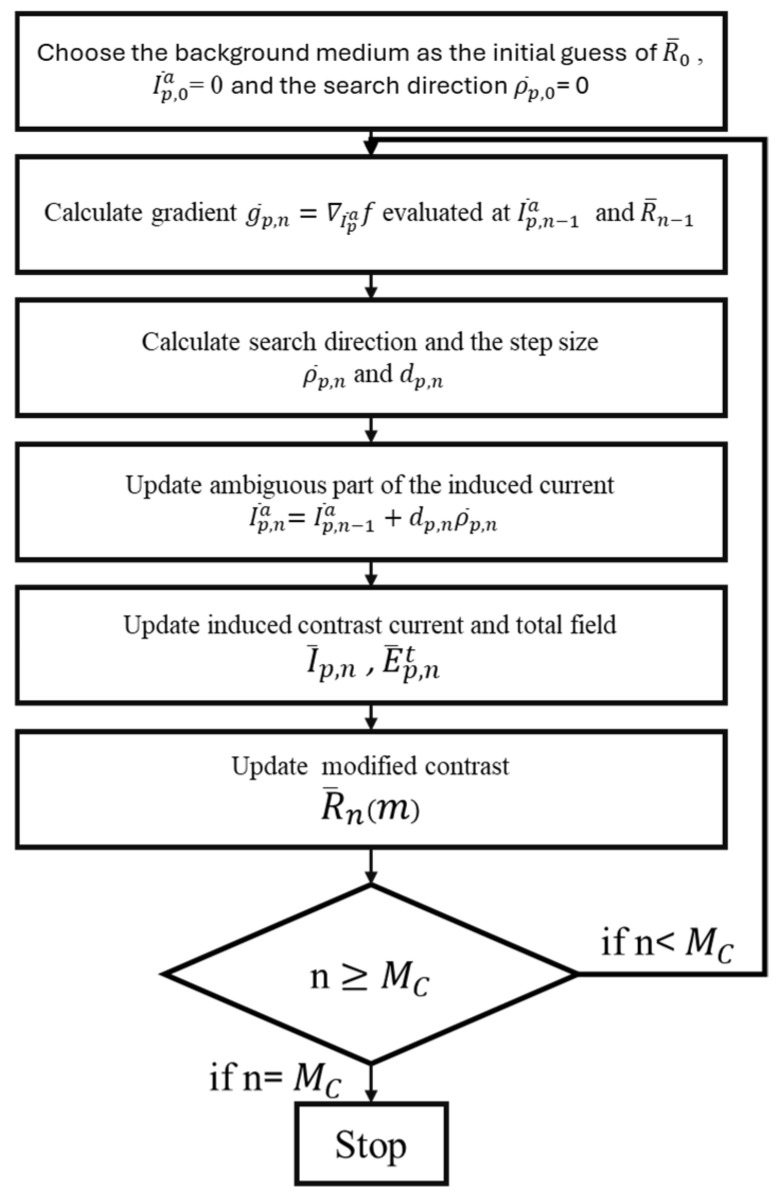
The flow chart of IMCS.

Figure 3 shows the flow chart of IMCS. When the number of iterations *n* meets the criticism, the iteration will stop. In our case, the iteration number is set to $M_{C}$. When we input the iteration result into U-Net as a preliminary image, $M_{C}$ is set to 3; when we use IMCS solely to reconstruct the image, $M_{C}$ is set to 200.

## 3. U-Net

**Figure 4:**
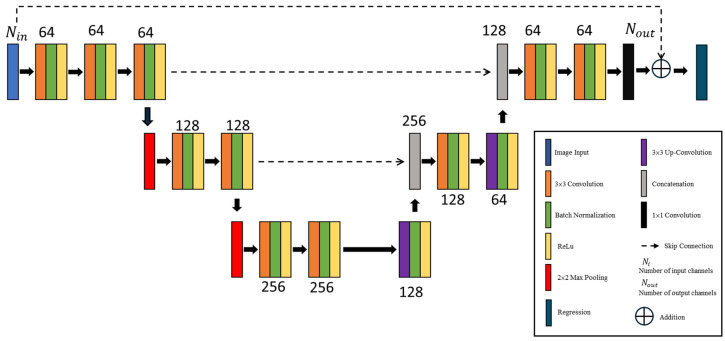
U-Net structure.

U-Net structure, as shown in Figure 4, has two main sections: a contracting path on the left and an expanding path on the right. The contracting path compresses the input by gradually reducing its spatial dimensions while capturing essential features. The expanding path then reconstructs the original dimensions by combining and refining these learned features to create a high-resolution output. In this structure, 3×3 convolution layers identify the features of the input image. By sliding convolutional filters across the image, they generate feature maps that highlight important details. Batch normalization layers bring stability to the learning process by normalizing the inputs. While ReLU layers introduce non-linearity, enhancing the network’s capability to identify complicated patterns and make precise predictions. To decrease the dimensions of the feature maps and avoid overfitting, pooling layers perform down-sampling by selecting the highest values within small, non-overlapping regions of each feature map. This step, accomplished through a 2×2 max-pooling layer in the contracting path, reduces both the data size and model parameters, facilitating faster training and enhancing efficiency. In the expanding path, spatial resolution is restored by an up-sampling layer using a 3×3 up-convolution, effectively reversing the down-sampling and bringing back finer details.

Throughout these processes, the number of input channels $N_{in}$ equals the number of output channels $N_{out}$, maintaining consistent feature depth. Lastly, a 1×1 convolutional layer serves as the decision-making layer, integrating all the extracted features to produce the final predictions. The outputs from this layer are averaged, and the mean result is passed to a regression layer to compute the error in predicting the permittivity distribution.

U-Net is widely utilized in image segmentation tasks for its ability to achieve high accuracy, retain fine details, and perform effectively even with limited information. Here are the primary reasons for choosing U-Net:

1. High precision in segmentation: U-Net is designed to achieve pixel-level segmentation, which is essential in fields like medical imaging, where precise boundary detection of structures (like organs or lesions) is critical. Its unique architecture captures both global context and local details, facilitating fine-grained segmentation.
2. Handle small and imbalanced datasets effectively: U-Net performs well even with small datasets, which is often the case in specialized fields such as healthcare. With its feature reuse and efficient learning structure, U-Net can generate accurate results without needing a vast amount of labeled data, thus lowering the cost and time involved in data annotation.
3. Skip connections for detail preservation: The skip connections in U-Net link the down-sampling (contracting) path to the up-sampling (expanding) path, reintroducing high-resolution information lost during down-sampling. This enables U-Net to retain fine details that are critical for distinguishing boundaries, which is particularly useful when segmenting small or intricate structures.
4. Multi-scale feature learning: By combining both low-level and high-level features through its down-sampling and up-sampling processes, U-Net can identify structures at different scales within the same image. This multi-scale approach enables robust segmentation, even for complex objects of varying sizes.
5. Computational efficiency: Despite being capable of delivering high-resolution outputs, U-Net is relatively lightweight and can run on modern GPUs, allowing faster training and real-time inference. This efficiency makes it perfect for applications that require both accuracy and fast performance.
6. Supports high-resolution output: U-Net can regenerate images efficiently, which is important for electromagnetic imaging, where segmented areas must precisely correspond to real anatomical structures.
7. Performance consistency across tasks: U-Net consistently performs well across segmentation tasks, from binary to multi-class segmentation, This makes it a dependable option for tasks that require accurate localization and boundary detection. In summary, U-Net is preferred for tasks requiring high-resolution and precise segmentation, particularly when input is limited and accuracy is essential. Its ability to capture multiple scales of information, retain high-level details, and perform efficiently makes it a powerful tool for image segmentation across a range of industries.

The minimization formula for dielectric constant $\varepsilon_{r}$ by U-Net is as follows:

(27) $${\left\|\Omega \left(\varepsilon_{r}^{M}\right)-\varepsilon_{r}\right\|}_{F}+\Lambda \left(\varepsilon_{r}\right)$$

where Ω are the U-Net architectures. $\varepsilon_{r}^{M}$ is the initial image by IMCS, $\varepsilon_{r}$ is the ground truth, and F signifies the Frobenius norm. Λ denotes the regularization function.

## 4. Numerical Results

A simulated model is created to conduct numerical analysis on inverse scattering for dielectric objects buried in half-space in this section. The dielectric constant for half-space $\varepsilon_{2}$ is 2.56. Our goal is to reconstruct the distribution of these objects’ relative permittivity. We achieve this by directing various TM-polarized plane waves into the region where the scatter is located and collecting detailed data on the scattered fields for reconstruction purposes. Regarding ADAM parameter settings, we establish the initial learning rate within the range of $10^{-3}$ to $10^{-5}$. The maximum epoch limit is set to 200. Furthermore, data shuffling is performed during each epoch of training. The performance of each solution is evaluated using the Normalized Root Mean Square Error (*NRMSE*) as follows:

(28) $$NRMSE=\frac{1}{M_{t}}\sum_{i=1}^{M_{t}}\frac{{\left\|\varepsilon_{r}-\varepsilon_{r}^{\alpha }\right\|}_{F}}{{\left\|\varepsilon_{r}\right\|}_{F}}$$

Let $\varepsilon_{r}^{\alpha }$ represent the reconstructed dielectric constant distribution, while $\varepsilon_{r}$ stands for the ground truth. The number of tests conducted is denoted by $M_{t}$.

We define the structural similarity index (*SSI*) as follows:

(29) $$SSI=\frac{\left(2\mu_{\tilde{y}}\mu_{y}+C_{1}\right)\left(2\sigma_{\tilde{y}y}+C_{2}\right)}{\left(\mu_{\tilde{y}}^{2}+\mu_{y}^{2}+C_{1}\right)\left(\sigma_{\tilde{y}}^{2}+\sigma_{y}^{2}+C_{2}\right)}$$

### 4.1. Relative Permittivity Between 4 and 6, with 5% Gaussian Noise

**Figure 5:**
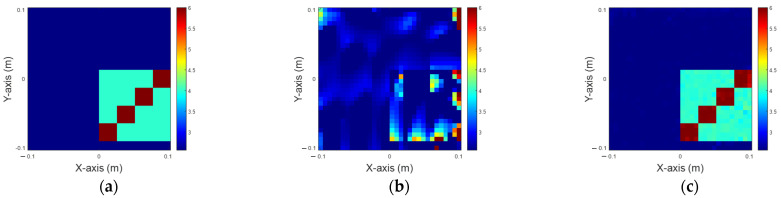
Reconstruction of permittivity between 4 to 6. (**a**) Ground truth; (**b**) IMCS with 200 iterations; (**c**) U-Net with three iterations.

In this simulation, the dielectric coefficient is distributed between 4 and 6. A total of 32 transmitters and 32 receivers are deployed, and 5% Gaussian noise is added separately. It is assumed that the scatterers have 10 distinct permittivity distributions, which can be situated at any of 50 different locations in an interested domain. Consequently, there are 500 images (10 × 50) for each scenario. The dataset is split into 80% for training and the remaining 20% for testing. Finally, we evaluate the results recovered solely by 200-iteration IMCS and the U-Net using the IMCS with three iterations, shown in Figure 5. Since IMCS has not converged at 100 iterations, it always converged at the 200th iteration in our simulation. As a result, we set the maximum number of iterations as 200. The NRMSE and SSI of the recovered images are depicted in Table 1. As observed, the dielectric permittivity within the inner region of the image reconstructed by IMCS with 200 iterations is blurred but is clearer in the U-Net application. The results reveal that U-Net performs better in reconstructing the permittivity distribution within the inner region of the image.

### 4.2. Relative Permittivity Between 6 and 8, with 5% Gaussian Noise

**Figure 6:**
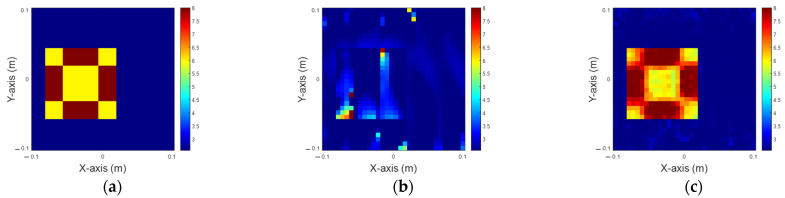
Reconstruction of permittivity between 6 to 8. (**a**) Ground truth; (**b**) IMCS with 200 iterations; (**c**) U-Net with three iterations.

In this scenario, the permittivity distribution is within the range of 6 to 8. Again, there are 32 transmitters and receivers in our simulation. We introduce 5% of Gaussian noise into the measured scattered field to emulate real-world conditions. The dataset is split into 80% for training and 20% for testing. Figure 6a shows the ground truth image. Figure 6b,c show the reconstructed image using solely IMCS with 200 iterations and U-Net combining IMCS with three iterations, respectively. The NRMSE and SSI are listed in Table 2. It is clear the inner part of the image is very blurred by IMCS with 200 iterations and is clearer by U-Net with IMCS for three iterations. Compared to Figure 4, it is evident that as the dielectric coefficient shifts from 4 and 6 to 6 and 8, there is a notable variation in both NRMSE and SSI, highlighting the sensitivity of these metrics to changes in the dielectric coefficient.

### 4.3. Relative Permittivity Between 8 and 10, with 5% Gaussian Noise

**Figure 7:**
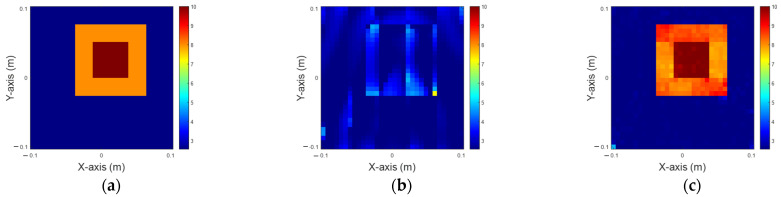
Reconstruction of permittivity between 8 to 10. (**a**) Ground truth; (**b**) IMCS with 200 iterations; (**c**) U-Net with three iterations.

In this scenario, we analyze the distribution of the dielectric constant in the range of 8 to 10, with 5% noise added. Figure 7a illustrates the ground truth, while Figure 7b,c present the reconstructed images obtained by 200 iterations IMCS alone and U-Net with the IMCS add-on after three iterations, respectively. The related NRMSE and SSI values are summarized in Table 3. It is seen that both the inner and outer parts of the dielectric constant are not good by IMCS with 200 iterations. However, the image is fair by U-Net with three-iteration IMCS. Comparing these results with Figure 5 and Figure 6, as the dielectric constant increases, the reconstructed object becomes progressively blurrier.

### 4.4. Relative Permittivity Between 4 and 6, with 5%, 10%, 15%, and 20% Gaussian Noise

**Figure 8:**
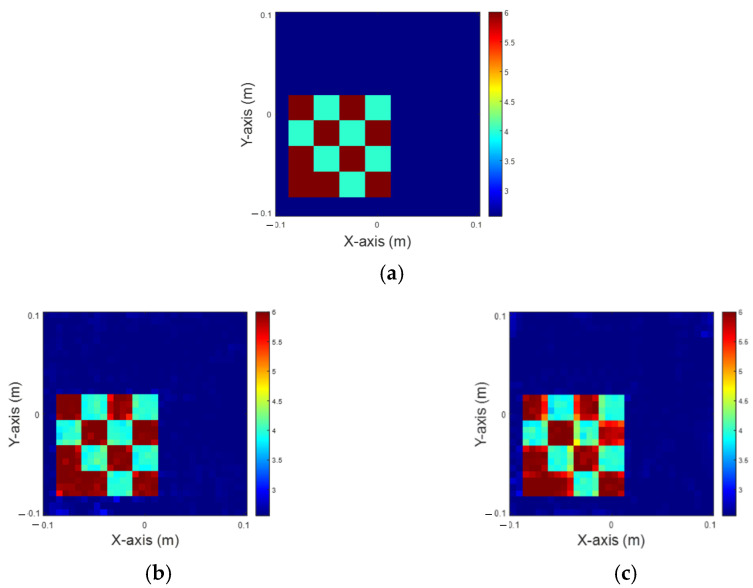

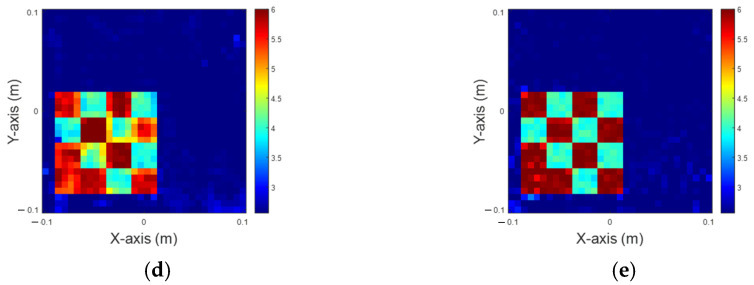
Reconstruction of permittivity 4 to 6. (**a**) Ground truth; (**b**) with 5% Gaussian noise; (**c**) with 10% Gaussian noise; (**d**) with 15% Gaussian noise; (**e**) with 20% Gaussian noise.

To replicate realistic scenarios, Gaussian noise levels of 5%, 10%, 15%, and 20% are added individually in our test scenario. As shown in Figure 8, the dielectric constant inside the reconstructed image becomes blurrier as the Gaussian noise increases. Figure 8 illustrates that as the Gaussian noise increases from 5% to 20%, the quality of the reconstructed images clearly deteriorates. Table 4. provides a summary of the corresponding NRMSE and SSI values. Higher noise levels, especially at 15% and 20%, introduce small blurring and artifacts, making it a little bit harder to distinguish object details. The reconstruction remains fairly accurate at 5% noise, but it becomes less reliable when the noise exceeds 20%. These results highlight the lower sensitivity of the reconstruction method to noise by U-Net.

### 4.5. Relative Permittivity at 6, with 5%, 10%, 15%, and 20% Gaussian Noise, Implementing IMCS Alone with 200 Iterations

**Figure 9:**
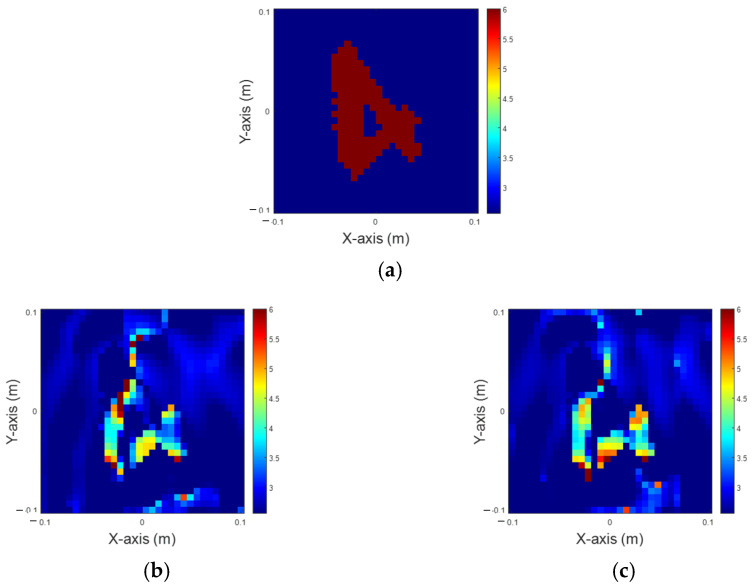

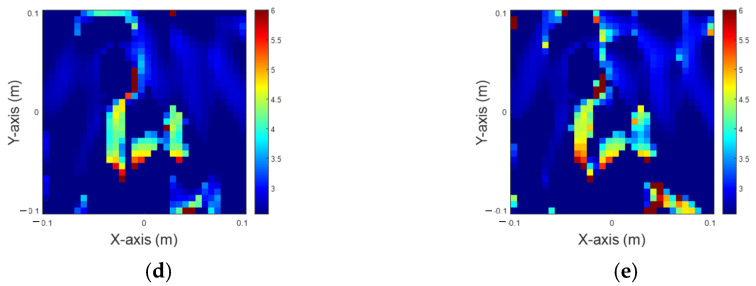
Reconstruction of permittivity at 6. (**a**) Ground truth; (**b**) with 5% Gaussian noise; (**c**) with 10% Gaussian noise; (**d**) with 15% Gaussian noise; (**e**) with 20% Gaussian noise.

Figure 9 shows the reconstruction results at different noise levels with a permittivity value of 6. It is found that the construction is not good by 200-iteration IMCS alone, even at 5% Gaussian noise. Numerical results indicate that as the noise level increases, the reconstruction quality deteriorates. NRMSE and SSI values are listed in Table 5.

### 4.6. Relative Permittivity at 6, with 5%, 10%, 15%, 20% Gaussian Noise, U-Net with 3 Iterations IMCS

**Figure 10:**
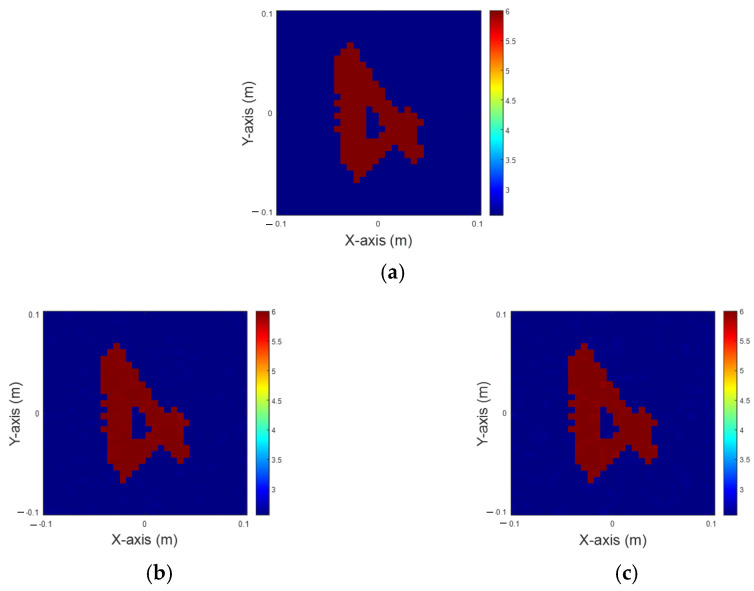

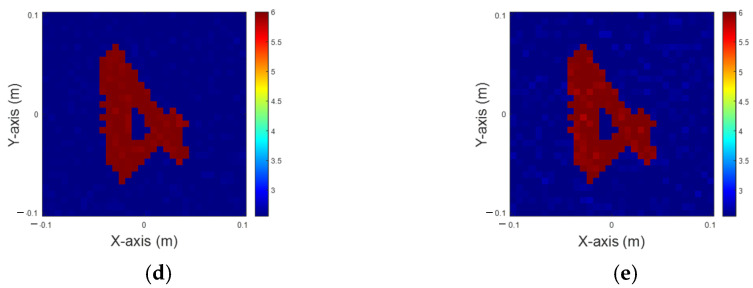
Reconstruction of permittivity at 6. (**a**) Ground truth; (**b**) with 5% Gaussian noise; (**c**) with 10% Gaussian noise; (**d**) with 15% Gaussian noise; (**e**) with 20% Gaussian noise.

Figure 10 shows the reconstruction results at different noise levels with a permittivity value of 6. It is found that the quality of the reconstructed image obviously deteriorates with the increase of Gaussian noise. However, compared to complex patterns, objects composed of a single dielectric coefficient are easier to restore. A detailed comparison of NRMSE and SSI values is presented in Table 6.

## 5. Conclusions

This paper presents the innovations of integrating U-Net with IMCS to solve the nonlinear challenges in electromagnetic imaging for buried objects in half-space. By utilizing a few iterations of the IMCS, the estimated initial images are generated. This approach greatly reduces both the calculated time for training and the computational burden for neural networks. Comparative results between standalone 200-iteration IMCS and the hybrid IMCS-U-Net approach with only three iterations show that IMCS–U-Net model obtains better images and enhanced noise resilience. Moreover, the method demonstrates strong noise resistance, even in environments with high dielectric constants. For dielectric constant ranges from 4 to 10, NRMSE and SSI are still good enough. This breakthrough may pave the way for future research exploring combinations of IMCS with sophisticated machine learning technologies, such as GANs and spatial and channel attention techniques, to tackle more intricate challenges. We can restore most objects; however, reconstructing complex objects in non-uniform backgrounds, such as the Austria profile, remains challenging. The quality declines significantly when noise exceeds 30%. In the future, we aim to improve reconstruction by utilizing multi-frequency techniques and hybrid inputs. Additionally, more advanced neural networks, such as generative adversarial networks, can be employed to further enhance reconstruction.

## Figures and Tables

**Table 1:** NRMSE and SSI for dielectric constants between 4 and 6.

	IMCS with 200 Iterations	U-Net with Three Iterations of IMCS
NRMSE	55.84%	1.17%
SSI	50.58%	85.75%

**Table 2:** NRMSE and SSI for dielectric constant between 6 and 8.

	IMCS with 200 Iterations	U-Net with Three Iterations of IMCS
NRMSE	58.6%	5.84%
SSI	42.34%	67.69%

**Table 3:** NRMSE and SSI for dielectric constant between 8 and 10.

	IMCS with 200 Iterations	U-Net with Three Iterations of IMCS
NRMSE	61.79%	6.1%
SSI	30.35%	63.8%

**Table 4:** NRMSE and SSI for permittivity 4 to 6 with different Gaussian noises.

	5%	10%	15%	20%
NRMSE	2.3%	2.8%	3.19%	6.74%
SSI	82.02%	80.4%	69.9%	63.6%

**Table 5:** NRMSE and SSI of IMCS for permittivity at 6 with different Gaussian noises.

	5%	10%	15%	20%
NRMSE	52.2%	52.4%	54.79%	65.18%
SSI	48.32%	49.42%	41.13%	40.2%

**Table 6:** NRMSE and SSI for permittivity at 6 with different Gaussian noises.

	5%	10%	15%	20%
NRMSE	0.568%	0.609%	0.892%	1.695%
SSI	88.05%	87.20%	79.46%	64.45%

## Data Availability

Data are contained within the article.
